# Supplementary material for: Chromothripsis during telomere crisis is independent of NHEJ, and consistent with a replicative origin
Source: Genome Res. 2019 May;29(5):737–49. doi: 10.1101/gr.240705.118 (PMC6499312; doi:10.1101/gr.240705.118)
Supplement: Supplemental Material [file supp_gr.240705.118_Supplemental_file_1.zip › contigs/annotated_contigs/DB80/contig.2.DB80_length_772_mean_cov_9.90932642487.docx]

**DB80_length_772_mean_cov_9.90932642487**

```
AACAAGCAACTATTGACAGGAAATTGAAAAAATTAAAAATTACCCTTGATGCCGACATGAGCCTCATTAGTGAGATTGAAGAACAATTT
 >chr5:84427663-84428021 - E=1e-203 p=1e-02
TCCAGCTTGATGAGGATGAGAGACAGAGTTTATTGGGTTGAATAATGAATACAAGATTAAAAAATGGAAACAGTAAGTATTGAAGTTTG

CTTTTGATAGGAGAGTACTTGTTACATTTTTAATAAAAGAGAAAATATGCAAAAGCCAATAGCAAATATGCAGATGCAGGGGGAAGAAG

GTTGATTATGTAAGAGAATAAGGATATCTAAAAGGATAAATTTACTAAGAGAGTAGAAGGGATAGACATGAAAACAAACATCAAACATA

A|A|AGCAGGCCCTCACAAGACATCAAATCTACTGGTGGCTTGATCGTGGACTTCCCTGGCTCAAGAACTGTCAGAAATACATTTCTGA
  >chr5:84425492-84425907 - E=8e-235
TCTATATAAGCCAGTCTATGGTAGTTTTAAGACAGAAATATACATGCTCATAATATACACATTGCAGTGAGCATGCGATTTTGTCTCTT

ATTCATTTATTATATGGAAAGTCTAGTATATGAAATAAGCTAATGCTCAGAAAGGTTTTAGTATACTGACTGACACATAATATATTTGT

AATAAATGTGAGCTAGAACAACAATTATGTTAGCTATAGAAGCTTTTCTCAATCAGGCATTCTTTCAACAATTTGTTGCGTTGAAAAGC

AAGATCAGCTTCTAGTTGTATATATTCTTTTTGCACTAAAATTGTTATTGCTGTCTGGACTA
```
